# To the Medical Profession of Texas

**Published:** 1896-04

**Authors:** 


### To the Medical Profession of Texas.

SHERMAN, TEXAS, March 31, 1896.

The undersigned committee, appointed at the last meeting of the Texas State Medical Association, "to take into consideration the recommendations of the president in his message, on the best means of increasing the membership, and thereby the usefulness of the State Medical Association;" also, to take into consideration "the legislation required in regard to medical matters, and devise ways and means by which the ends sought may be reached," after considerable labor, and a thorough discussion in a conference of the questions before us, have formulated a report, to be presented to the next meeting of the Association at Fort Worth. We believe that a careful consideration of and wise action upon the recommendations in this report by that body will result in estimable good to the Association, to all the people of Texas, to individual members of the profession, to the honor, dignity and usefulness of organized medicine in its scientific aspects, in its progress as an art and a science, and help to place it upon the high plane among the learned professions to which it belongs. There is a vast field in this great empire State unexplored by medical research and scientific investigation. There is ability and talent in the profession of the State, of which it can well afford to be proud, but it is not yet properly utilized. Its State organization, as a great scientific and influential body, should be the peer of any in the Union. The medical legislation, so long and sadly needed, to protect our people from the ravages of pestilence from ignorance and quackery, the better supervision and welfare of the unfortunate of our land, can all be readily obtained if carefully and properly presented in legal and scientific form, with the recommendations of a united and harmonious profession.

We, therefore, do earnestly appeal to every medical man in the State, who honors his prosession, who loves his people, who has a conscientious regard for their welfare, who desires the promotion and progress of science, who feels the great necessity for improved State medicine and sanitary laws, and who desires his own advancement and firmer professional standing, with a just pride for a higher condition of medical matters in his State, to turn aside all obstacles, and meet the Texas State Medical Association at Fort Worth on the fourth Tuesday in April with a conscientious determination to aid this committee in its important work, and place the Lone Star State on the great medica plane to which it should belong. We expect and hope to see the largest gathering of medical men at this meeting that ever assembled in the State for any purpose.

Thos. D. Wooten,
J. F. Y. Paine,
M. D. Knox,
Bacon Saunders,
J. T. Wilson,
Committee.

